# Using Structural Equation Modeling to Assess the Links between Tobacco Smoke Exposure, Volatile Organic Compounds, and Respiratory Function for Adolescents Aged 6 to 18 in the United States

**DOI:** 10.3390/ijerph14101112

**Published:** 2017-09-25

**Authors:** Bonnie E. Shook-Sa, Ding-Geng Chen, Haibo Zhou

**Affiliations:** 1 Department of Biostatistics, University of North Carolina, Chapel Hill, NC 27599, USA; dinchen@email.unc.edu (D.-G.C.); zhou@bios.unc.edu (H.Z.); 2 School of Social Work, University of North Carolina, Chapel Hill, NC 27599, USA

**Keywords:** asthma, volatile organic compounds, tobacco, structural equation modeling

## Abstract

Asthma is an inflammatory airway disease that affects 22 million Americans in the United States. Research has found associations between impaired respiratory function, including asthma and increased symptoms among asthmatics, and common indoor air pollutants, including tobacco smoke exposure and volatile organic compounds (VOCs). However, findings linking VOC exposure and asthma are inconsistent and studies are of mixed quality due to design limitations, challenges measuring VOC exposure, small sample sizes, and suboptimal statistical methodologies. Because of the correlation between tobacco smoke exposure and VOCs, and associations between both tobacco smoke and VOCs with respiratory function, it is crucial that statistical methodology employed to assess links between respiratory function and individual air pollutants control for these complex relationships. This research uses Structural Equation Modeling (SEM) to assess the relationships between respiratory function, tobacco smoke exposure, and VOC exposure among a nationally-representative sample of adolescents. SEM allows for multiple outcome variables, the inclusion of both observed and latent variables, and controls the effects of confounding and correlated variables, which is critically important and is lacking in earlier studies when estimating the effects of correlated air pollutants on respiratory function. We find evidence of associations between respiratory function and some types of VOCs, even when controlling for the effects of tobacco smoke exposure and additional covariates. Furthermore, we find that poverty has an indirect effect on respiratory function through its relationships with tobacco smoke exposure and some types of VOCs. This analysis demonstrates how SEM is a robust analytic tool for assessing associations between respiratory function and multiple exposures to pollutants.

## 1. Introduction

Asthma is an inflammatory airway disease that affects 22 million Americans and is the most common chronic disease in young children. The incidence of asthma has been growing over the past several decades [1,2]. This increase coincides with changes to indoor environments [2], and some researchers have attributed the increase to changes in environmental influences [3]. Asthma is a growing public health concern because of its prevalence, significant health risks, and high healthcare costs [3].

Impaired respiratory function, including asthma and increased asthma symptoms among asthmatics, has been linked to exposure to environmental endotoxins and common indoor air pollutants, including tobacco smoke and volatile organic compounds (VOCs) [1,2,4,5]. Air pollution is also associated with heightened susceptibility to respiratory infections among persons with allergies, and respiratory viral infections commonly exacerbate asthma [6]. The literature shows that both smoking and VOC exposure disproportionately affect persons living in poverty. Smoking rates are higher in lower-income areas than higher-income areas [7]. Exposure to VOCs disproportionately affects both persons living in poverty and children [8].

The literature linking smoking to asthma and asthmatic symptoms is extensive. Asthmatic symptoms are higher among asthmatics who smoke compared to those who do not smoke, and smokers experience higher morbidity rates due to asthma compared to non-smokers [1]. In addition, asthmatic children exposed to secondhand smoke have more severe disease symptoms compared with those who are not exposed to secondhand smoke, and these effects are greater for children exposed to multiple household smokers [1].

VOCs are chemical irritants that are contained in common household products including solvents, adhesives, paint, cleaning products, furniture, and air fresheners [2]. VOCs evaporate at room temperature from these products and are inhaled as a form of indoor air pollution. Three common types of VOCs, classified as aromatics, are xylenes, ethylbenzene, and toluene [9]. Xylenes and ethylbenzene are C8 aromatic isomers and are commonly combined into “mixed xylenes”. They have similar molecular structures and are used in motor gasoline and paint solvents [10]. Xylenes are also used in the printing, rubber, and leather industries and as a cleaning agent and thinner for paints and varnishes. Ethylbenzene is also found in inks and insecticides. Both xylenes and ethylbenzene are also contained in cigarette smoke [11,12]. Toluene is contained in gasoline and fuels as well as paints, paint thinners, fingernail polish, and adhesives [13].

Concentrations of VOCs are five to ten times higher in indoor environments than outdoor environments [2]. Children are exposed to high levels of VOCs because they spend the majority of their time indoors [3]. VOCs are higher in households with smokers [3]. A risk assessment conducted on toluene, ethylbenzene, and xylene in consumer products found that common exposure to these chemicals exceeds safe limits and needs to be reduced [14].

There is substantial literature showing associations between exposure to VOCs, including xylene, ethylbenzene, and toluene, with asthma, asthmatic symptoms, and impaired respiratory function [2,4,9,15]. However, systematic reviews note that findings are inconsistent and that many of the findings are based on studies of poor quality due to design limitations, challenges measuring VOC exposure, small sample sizes, and suboptimal statistical methodologies [9,15,16]. These researchers have called for more robust studies such as case-control studies and randomized controlled trials to assess causal links between VOCs and respiratory impairment [9,15,16]. Because exposure to VOCs can be reduced (e.g., by changing to low-VOC furniture, improving ventilation, avoiding perfumed items), it is crucial to understand if and how these chemicals contribute to respiratory function [3].

Assessing the links between indoor air pollutants and respiratory function presents multiple analytic challenges that must be solved in order to provide robust findings. The methodology employed must control for the effects of multiple correlated air pollutants and must be able to estimate the effects of each individual pollutant. Furthermore, there is no single measure that captures all elements of respiratory function, so it is preferable to be able to model multiple endpoints simultaneously. This paper assesses the relationship between tobacco smoke exposure, VOCs, and impaired respiratory function among adolescents in the United States within a structural equation modeling (SEM) framework using nationally-representative data. SEM is a statistical methodology that uses a series of regression equations to model relationships that can be depicted pictorially [17]. SEM provides a more robust analysis that offers several advantages in modeling the associations between these indoor air pollutants and respiratory function. It is a compelling analytic tool for exposure analyses because it has the capability to model multiple outcomes simultaneously, accommodates the inclusion of both observed and latent variables, allows for the analyses of highly correlated data, incorporates measurement error, and allows for the estimation of indirect effects [18]. In addition, regressions can be included not only from predictors to outcome variables but also between multiple predictor variables. It allows flexibility in including covariates that are associated with only one exposure as these covariates help estimate individual latent variables [18]. While SEM has been used to model inflammatory response to traffic air pollution [19], no literature could be found using SEM to assess links between indoor air pollution and respiratory function. The aim of this study is to use SEM to better understand the relationships between indoor air pollutants and respiratory function while demonstrating how SEM can be used as a robust technique for multi-pollutant modeling.

## 2. Materials and Methods

Data for this analysis came from the 2011–2012 National Health and Nutritional Examination Surveys (NHANES). The NHANES is a nationally-representative in-person survey of the noninstitutionalized civilian resident population of the United States that focuses on health and nutrition. It includes detailed questionnaires regarding health conditions and behaviors and a health evaluation that is conducted in a mobile examination center (MEC). In the MEC, participants receive a physical exam and urine and blood samples are collected. All persons within selected households are eligible to participate, with proxy interviews conducted for adolescents as needed [20].

The NHANES includes a number of items regarding respiratory function, asthma, and other respiratory diseases, including physical assessments and self-reported data from respondents. Of interest in this analysis are three spirometry measures of lung function: peak expiratory flow (PEF), extrapolated volume (NEV), and forced vital capacity (FVC). These are common pulmonary function indicators measured as part of the spirometry exam conducted in the MEC [21]. PEF, NEV, and FVC measure the rate and volume of air an individual can expire from his/her lungs and are commonly used in the diagnosis of respiratory diseases, including asthma. Lower levels of PEF, NEV, and FVC are associated with impaired respiratory function [21]. It is important to note, however, that spirometry readings vary by age, height, and weight, so it is expected that younger persons and persons with a small body size have lower spirometry readings regardless of respiratory health [22,23]. Expert consultants working for the Centers for Disease Control and Prevention review, process, and edit all spirometry measures to ensure internal data consistency prior to the release of the NHANES public use file [21].

NHANES contains questionnaire items regarding tobacco use by the respondent and tobacco exposure in the respondent’s home. Because this analysis is focused on adolescents, secondhand smoke is the primary tobacco exposure of interest. Furthermore, personal smoking behavior was only collected for persons aged 12 and over, so personal smoking could not be included for persons under 12. The average number of cigarettes smoked in the respondent’s home per day and the number of smokers in the respondent’s household were used to quantify secondhand smoke exposure. Inclusions of these measures is consistent with findings that children with multiple household smokers have increased risk of respiratory effects [1].

In the 2011–2012 NHANES, metabolites for volatile organic compounds (VOC) were examined in the urine samples of one third of NHANES participants aged 6 years and older. A crosswalk provided in the laboratory procedures manual was used to map the VOC metabolites to their parent compounds: Xylenes are 2-Methylhippuric acid, 3-methipurc acid, and 4-methipurc acid; Ethylbenzene is Phenylglyoxylic acid; and Toluene is *N*-Acetyl-*S*-(benzyl)-l-cysteine [24]. Xylene, ethylbenzene, and toluene were used in subsequent analyses because of their suspected associations with asthma and asthmatic symptoms.

Finally, because of their associations with smoking, VOCs, and/or respiratory function, the covariates age, poverty status, height, and weight were included in the analysis. Age and poverty status were based on survey items, while height and weight were measured during the MEC examination. The NHANES calculates poverty as the ratio of the participant’s family income to the Department of Health and Human Services’ poverty threshold (specific to the participant’s household size and geographic location) [25]. Thus, a higher poverty ratio is associated with higher income relative to the poverty threshold (i.e., lower levels of poverty).

Of the 9756 respondents in the 2011–2012 NHANES, those who did not receive the VOC testing were excluded (including all participants five years and younger as VOC testing was only conducted for persons aged 6 and over). In addition, the analysis was restricted to adolescents (aged 6 to 18). This lead to a total sample size of 748 respondents. The different VOC metabolites had quite different ranges and variances, as did the different spirometry measures. For this reason, VOC metabolites and spirometry measures were standardized prior to the analysis to facilitate model fitting. Because this is a nationally-representative sample, these data are representative of adolescents in the United States aged 6–18 years. This sample should allow for a reliable assessment of the associations between tobacco smoke exposure, VOCs, and respiratory function in the general population of adolescents in the United States.

After limiting the dataset and standardizing the variables as described, Pearson’s correlation coefficients were calculated and principal components analyses were performed within the respiratory function, tobacco, and VOC groups of variables. We measured respiratory function with a single factor which included all three spirometry measures. A single factor, consisting of the two secondhand smoke measures, was used to estimate tobacco smoke exposure. The VOC measurement group was split into two factors, with the two xylene metabolites and ethylbenzene comprising one factor and toluene as its own factor. This is consistent with the literature because, as previously discussed, xylene and ethylbenzene are both C8 isomers with similar molecular forms and are often mixed during production [10].

Following the establishment of well-fitting measurement models of the three latent variables (tobacco exposure, xylene/ethylbenzene, and respiratory function), a SEM was fit to assess the relationships between respiratory function, tobacco smoke exposure, and VOCs among adolescents, controlling for covariates (age, height, weight, and poverty status). Model fitting was conducted using Mplus software (model details are provided in Section 3.2). Both the measurement models and SEMs were fit using the Maximum Likelihood Robust (MLR) estimator to account for non-normally distributed data and the small amount of missing data [17]. The MLR estimator adjusts standard errors using a Huber–White sandwich estimator and scales fit statistics to account for non-normality of observed variables. This estimator accommodates missing completely at random or missing at random data patterns [26]. The subsample analytic weights were used in all analyses so that estimates are nationally-representative, as these weights account for the multi-stage sample design and subsampling for VOC testing. A final parsimonious model was fit by dropping weak associations in the original SEM, and the final model was used to assess the relationships between VOCs and respiratory function, controlling for the effects of tobacco smoke exposure on both VOCs and respiratory function. Finally, both the direct and indirect effects of poverty on respiratory function were assessed.

## 3. Results

### 3.1. Descriptive Statstics

Descriptive statistics for the NHANES variables used in this analysis are included in Table 1. Most variables had fairly low missing rates, ranging from 0% to 7.6% missing. All variables were non-normally distributed, leading to the use of the MLR estimator in subsequent model fitting.

Weighted Pearson correlation coefficients between each pair of observed variables within the three latent variables are presented in Table 2. Observations were weighted with the subsample analytic weights so that the estimates are nationally-representative. Within the tobacco latent variable, the two observed secondhand smoking variables are highly correlated (r = 0.865). The xylene/ethylbenzene observed variables are also correlated, with the highest correlation between xylene1 and xylene2 (r = 0.692) and the lowest correlation between ethylbenzene and xylene1 (r = 0.172). The respiratory function latent variable demonstrates high levels of correlation within its observed variables, with PEF and FVC demonstrating the highest correlation (r = 0.877) and PEF and NEV demonstrating the lowest correlation (r = 0.495). The consistency of the observed variables within latent variables is further assessed in the fitting of a subsequent measurement model.

### 3.2. Model Fitting

**Figure 1:**
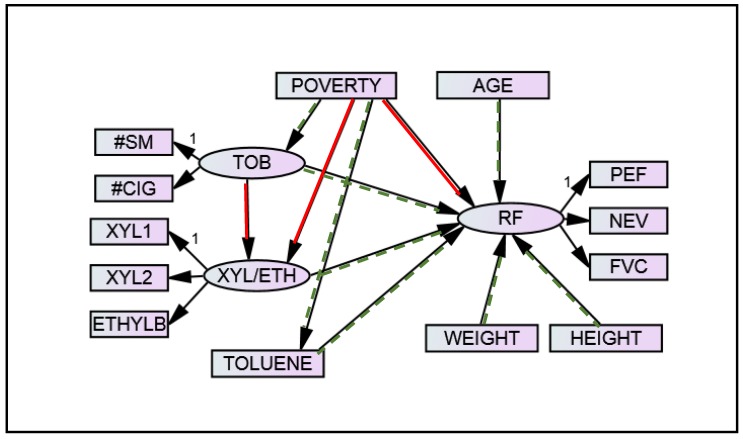
Hypothesized SEM of respiratory function, tobacco smoke exposure, and VOCs. This model predicts respiratory function based on tobacco smoke exposure and VOCs (xylene/ethylbenzene and toluene), controlling for the effects of age, height, weight, and poverty status on respiratory function and poverty status on tobacco smoke exposure and VOCs. Green (dotted) paths are significant at the *α* = 0.15 level; red (solid) paths are not significant at the 0.15 level. “1” identifies the observed variable within each latent variable whose factor loading was fixed to 1.

As shown in Figure 1 and represented by Equations (1a–e) and (2a–c) below (syntax modeled after [18]), the hypothesized measurement model included three latent variables for respiratory function, tobacco smoke exposure, and xylene/ethylbenzene exposure. Toluene (Equation (1f)) is included in the SEM directly as an observed variable, and is omitted from the measurement model. Respiratory function was measured through spirometry measures (FVC, NEV, and PEF), with lower values corresponding to poor respiratory function and higher values corresponding to higher function (Equation (2a–c)). Tobacco smoke exposure (Equation (1a,b)) was measured through the two survey items of secondhand smoke exposure (number of cigarettes smoked in the household per day, on average) and the number of smokers in the household. The xylene/ethylbenzene latent variable was measured with two xylene measures and ethylbenzene, as these VOCs are closely related (Equation (1c–e)). This model serves solely to establish a well-fitting measurement model of the three latent variables upon which the subsequent SEM can be built.

Modeling Predictor Variables:

(1a) $$X_{1}=\upsilon_{x1}+\lambda_{11}L_{1}+\varepsilon_{x1}$$

(1b) $$X_{2}=\upsilon_{x2}+\lambda_{21}L_{1}+\varepsilon_{x2}$$

(1c) $$X_{3}=\upsilon_{x3}+\lambda_{31}L_{2}+\varepsilon_{x3}$$

(1d) $$X_{4}=\upsilon_{x4}+\lambda_{41}L_{2}+\varepsilon_{x4}$$

(1e) $$X_{5}=\upsilon_{x5}+\lambda_{51}L_{2}+\varepsilon_{x5}$$

(1f) $$X_{6}=\upsilon_{x6}+\lambda_{61}C_{1}+\varepsilon_{x6}$$

Modeling Outcome Variables:

(2a) $$Y_{1}=\upsilon_{y1}+\;\lambda_{12}L_{3}+\varepsilon_{y1}$$

(2b) $$Y_{2}=\upsilon_{y2}+\;\lambda_{22}L_{3}+\varepsilon_{y2}$$

(2c) $$Y_{3}=\upsilon_{y3}+\;\lambda_{32}L_{3}+\varepsilon_{y3}$$

where *L*_1_, *L*_2_, and *L*_3_ are the tobacco, xylene/ethylbenzene, and respiratory function latent variables, respectively; *X*_1_ and *X*_2_ are the observed tobacco variables (number of smokers in the household and average number of cigarettes smoked in the home); *X*_3_, *X*_4_, and *X*_5_ are the xylene/ethylbenzene observed variables (xylene1, xylene2, and ethylbenzene); *X*_6_ is toluene; *Y*_1_, *Y*_2_, and *Y*_3_ are the observed respiratory function variables (PEF, NEV, and FVC); *C*_1_, is the covariate poverty; $\upsilon_{i}$ are intercept terms; $\lambda_{i}$ are regression coefficients; and $\varepsilon_{i}$ are error terms.

When fitting this initial model, the residual covariance matrix was not positive definite. Upon examining the number of smokers in the household variable, the residual variance was very small and non-significant, but negative. Obtaining a negative residual variance during model fit can be caused by a small sample size, model misspecification, or skewed data (e.g., floor effects). In this case, the number of smokers in the household is a highly skewed variable, resulting in this slightly negative residual variance. This residual variance was set to zero to allow the model to fit appropriately. The subsequent measurement model terminated normally and exhibited a very good fit. The scaled chi-squared test statistic for the likelihood-ratio test is 34.693 with 18 degrees of freedom, leading to a *p*-value of 0.0103. However, this goodness of fit test is often rejected in SEM because of its sensitivity to large sample sizes [17]. The comparative fit index (CFI) [17,27] and Tucker Lewis index (TLI) [28] values are 0.961 and 0.940, respectively, and a rooted mean square error of approximation (RMSEA) is 0.035. CFI and TLI are two common measures of fit in SEM that measure the proportion of improvement in model fit of the hypothesized model with a baseline model that is less restrictive. CFI and TLI values close to 1.0 (preferably over 0.95) indicate a well-fitting model. RMSEA is commonly used in SEM as an absolute measure of fit, and estimates how well the model fits the population covariance matrix. RMSEA values below 0.05 are indicative of good fit [17]. Because this model does not yet include toluene and the covariates age, height, and weight, and poverty status, parameter estimates are not useful. However, this model demonstrates that the construction of latent variables is reasonable and allows for the development of a more complex SEM.

The hypothesized SEM is depicted in Figure 1 and is represented by the equations for the measurement model above in addition to the structural equations. The hypothesized SEM includes the three latent variables fit in the measurement model (Equations (1a–e) and (2a–c) above), with the tobacco and xylene/ethylbenzene latent variables predicting respiratory function (Figure 1; Equation (3c) below). Because tobacco smoke contains xylene/ethylbenzene, a path from tobacco smoke exposure to xylene/ethylbenzene is included (Equation (3b)). Toluene is included as an observed variable, also predictive of respiratory function (Equations (1f) and (3c)). Poverty status predicts both tobacco smoke exposure and VOCs in the hypothesized model (Equations (1f) and (3a,b)). Poverty is also included as a direct predictor for respiratory function so that it can be determined if aspects of poverty apart from tobacco and VOC exposure influence respiratory health or, alternatively, if the effects of poverty on respiratory health are explained solely by tobacco and VOC exposure (Equation (3c)). Finally, age, height, and weight are included as predictors of respiratory function, as lung capacity expands through adolescence and for persons with larger body size (Equation (3c)). This controls for potential confounding effects when measuring the other associations.

Modeling the Latent Variables:

(3a) $$L_{1}=\alpha_{1}+\;\beta_{11}C_{1}+\xi_{1}$$

(3b) $$L_{2}=\alpha_{2}+\;\beta_{21}L_{1}+\;\beta_{22}C_{1}+\xi_{2}$$

(3c) $$L_{3}=\alpha_{3}+\;\beta_{31}L_{1}+\beta_{32}L_{2}+\;\beta_{33}X_{6}+\beta_{34}C_{1}+\beta_{35}C_{2}+\beta_{36}C_{3}+\beta_{37}C_{4}+\xi_{3}$$

where *L*_1_, *L*_2_, and *L*_3_ are the tobacco, xylene/ethylbenzene, and respiratory function latent variables, respectively; *X*_6_ is toluene; *C*_1_, *C*_2_, *C*_3_, and *C*_4_ are the covariates poverty, age, weight, and height, respectively; $\alpha_{i}$ are intercept terms; $\beta_{i}$ are regression coefficients; and $\xi_{i}$ are error terms.

The hypothesized SEM is fit in Mplus. As with the measurement model, the residual variance for the number of smokers in the household is constrained to zero to obtain convergence. The model exhibits reasonable fit, with a CFI and TLI of 0.946 and 0.928, respectively, and a RMSEA of 0.041. The scaled chi-squared test statistic for the likelihood-ratio test is 117.304 with 54 degrees of freedom, leading to a *p*-value of less than 0.0001. Figure 1 includes highlighting to indicate the significance level for paths in the hypothesized SEM. Those with green (dotted) arrows are significant at the *α* = 0.15 level, while red (solid) paths are not significant at the *α* = 0.15 level. An *α* value of 0.15 is used in Figure 1 to facilitate the development of a more parsimonious final model while maintaining potential marginally significant relationships. All relationships are in the direction expected based on theory. Higher levels of indoor air pollution (tobacco and VOCs) are associated with lower respiratory function. A lower ratio of family income to poverty (i.e., higher poverty) is associated with higher exposure to tobacco and VOCs. Tobacco exposure and xylene/ethylbenzene are positively associated, as is respiratory function with age, height, and weight. The path between tobacco exposure and xylene/ethylbenzene levels had a *p*-value of 0.177, and poverty was not an important predictor for xylene/ethylbenzene (*p*-value = 0.786) or respiratory function (*p*-value = 0.521).

**Figure 2:**
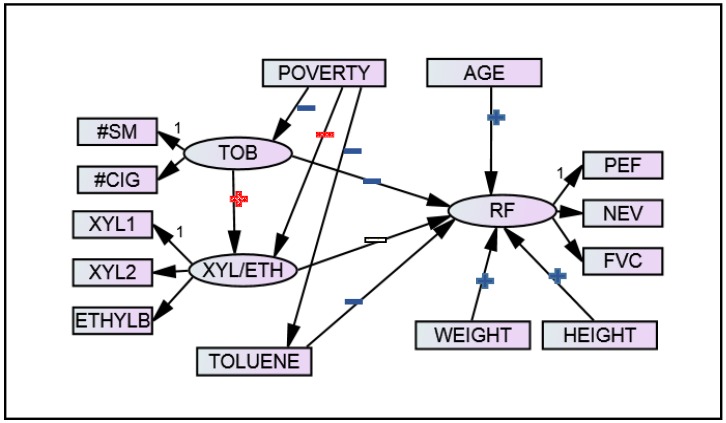
Final SEM of respiratory function, tobacco smoke exposure, and VOCs with significance and direction indicators. Plus signs indicate positive relationships and minus signs indicate negative relationships. Blue (solid) symbols are significant at the *α* = 0.05 level, white (unfilled) symbols are significant at the *α* = 0.15 level, and red (checkered) symbols are not significant at the 0.15 level. “1” identifies the observed variable within each latent variable whose factor loading was fixed to 1.

To provide a more parsimonious model, the path between poverty and respiratory function was removed from the final model, modifying Equation (3c) to Equation (3d) as represented below. This path was more exploratory, as theory indicated that poverty is a predictor of tobacco smoke exposure and VOCs, not as a direct predictor of respiratory function. This path also had a very large *p*-value (0.521). All other paths were maintained in the final model because these relationships were of primary interest in this analysis, so it was preferable to leave them in the final model to assess in the context of the other variables in the model regardless of their *p*-values in the initial hypothesized model. The parsimonious model is depicted in Figure 2 and includes Equations (1a–f), (2a–c), (3a,b), and (3d)

(3d) $$L_{3}=\alpha_{3}+\;\beta_{31}L_{1}+\beta_{32}L_{2}+\;\beta_{33}X_{6}+\beta_{34}C_{2}+\beta_{35}C_{3}+\beta_{36}C_{4}+\xi_{3}$$

where *L*_1_, *L*_2_, and *L*_3_ are the tobacco, xylene/ethylbenzene, and respiratory function latent variables, respectively; *X*_6_ is toluene; *C*_2_, *C*_3_, and *C*_4_ are the covariates age, weight, and height, respectively; $\alpha_{3}$ is an intercept term; the $\beta_{i}$ are regression coefficients; and $\xi_{3}$ is an error term.

Model fit was similar to the hypothesized model, with CFI and TLI values of 0.947 and 0.930, respectively, and an RMSEA of 0.041. The scaled chi-squared test statistic for the likelihood-ratio test is 117.158 with 55 degrees of freedom, leading to a *p*-value of less than 0.0001. Figure 2 indicates the direction and significance level of each association in this final SEM, with beta estimates, standard errors, and *p*-values for all paths presented in Table 3.

In addition, the indirect associations between poverty and respiratory function were measured to assess their significance. The *β* estimates, standard errors, and *p*-values for these indirect relationships are presented in Table 4.

## 4. Discussion

SEM is a useful approach for modeling the associations between indoor air pollutants and respiratory function. Because of the correlation between tobacco smoke exposure and VOCs, and associations between both tobacco smoke and VOCs with respiratory function, it is crucial that models seeking to understand the associations between individual indoor air pollutants and respiratory function control for these relationships. Researchers are increasingly recommending multi-pollutant modeling approaches rather than focusing on risks of single pollutants [29], and SEM lends itself to a multivariate approach. SEM can be used to effectively model not only the associations between indoor air pollutants and respiratory function, but can also control for the relationships among different types of indoor air pollutants and their related covariates. Because SEM accommodates latent variables, multiple correlated predictors and outcomes can be included in the measurement of tobacco smoke exposure, VOCs, and respiratory function. This robust analytic approach, when applied to nationally-representative data, provides strong evidence of the presence or absence of associations between indoor pollutants and respiratory function.

In this analysis, SEM served as a robust methodology for assessing the effects of multiple indoor air pollutant exposures on respiratory function among adolescents. Well-fitting measurement models and SEMs were established to model the associations between respiratory function, tobacco smoke exposure, VOC exposure, and covariates of interest. Because SEM was used, these models can assess the relationships between VOCs and respiratory function while controlling for the effects of tobacco smoke exposure both on VOC levels and on respiratory function. Furthermore, these models allow for multiple outcome variables, account for measurement error, and control for confounding variables: the effects of age, height, and weight on respiratory function and the effects of poverty status on VOCs and tobacco smoke exposure.

Our analysis of NHANES data using a SEM approach supports the hypothesis that respiratory function among adolescents is negatively associated with exposure to VOCs when controlling for the other variables in the model. The relationship is marginally significant for xylene/ethylbenzene (*p*-value = 0.120), while the association between toluene and respiratory function is more highly significant (*p*-value = 0.050). The effect of tobacco smoke exposure on xylene/ethylbenzene exposure was positive, but had a *p*-value of 0.177.

Poverty status had direct effects on tobacco smoke exposure (*p*-value less than 0.001) and toluene (*p*-value = 0.023), but the effect on xylene/ethylbenzene had a *p*-value of 0.781. This provides additional evidence that adolescents in poverty are exposed to higher levels of tobacco smoke and some VOCs (toluene). The indirect effect of poverty on respiratory function provides strong evidence that higher poverty is associated with lower respiratory function. This indirect relationship is driven primarily by the mapping of poverty to respiratory function through tobacco smoke exposure (*p*-value = 0.033). Adolescents in poverty are more likely to be exposed to secondhand smoke which, in turn, negatively affects respiratory function. Poverty also has a marginally significant indirect relationship on respiratory function through toluene (*p*-value = 0.097). Adolescents in poverty have higher exposure to toluene than those not in poverty, and this exposure is associated with poor respiratory function. This analysis provided evidence that poverty has an indirect effect on respiratory function that is explained through its relationships with tobacco smoke exposure and VOCs.

There are limitations associated with this analysis. The use of latent variables comprising standardized observed variables was effective in allowing for multiple endpoints and establishing whether or not there is evidence of a relationship between a class of VOCs and respiratory impairment. The limitation of using latent variables in this analysis is that the magnitudes of *β* estimates are not meaningful (i.e., one cannot use the model results to assess how different levels of VOC exposure in ng/mL contribute to declines in PEF, NEV, or FVC spirometry measures in mL or mL/s). Additionally, the VOC metabolites and respiratory function tests were reflective of the participants’ exposures to VOCs and lung function at the time of the medical exam in the MEC. However, respiratory function can be impacted by prior exposures that are not measured in the assessments conducted at the MEC. Furthermore, self-reported household smoking measures are subject to potential social-desirability effects and recall bias. Finally, while this analysis is strengthened by the use of a robust analytic technique (SEM) and a nationally-representative dataset from a methodologically-sound survey, the results are not based on a randomized controlled trial. As with observational studies, confounding variables not accounted for in the model could potentially explain observed associations. Some observed associations achieved only marginal significance, indicating the need for an even larger sample to better understand the marginally-significant relationships in the model. Despite these limitations, this model provides meaningful insight into the relationships between respiratory function, VOCs, and tobacco smoke exposure.

Future analyses can use SEM to model other indoor air pollutants simultaneously, including exposure to formaldehyde and perfluorinated compounds. Other asthma contributors, including exposure to dust and outdoor pollutants, could be included in a single comprehensive model. To further emphasize findings of other researchers, a randomized controlled trial would provide the most robust causal evidence of relationships between indoor air pollutants and respiratory function, regardless of the analytic technique used (e.g., testing interventions to reduce air pollutant exposure).

## 5. Conclusions

SEM allows complex relationships between predictor and outcome variables to be modeled, accommodates the inclusion of both latent and observed variables, and controls for relationships between multiple predictor variables. These strengths make SEM a robust analytic technique that can be used effectively to model exposure to multiple pollutants simultaneously while controlling for covariates of interest. In exposure analyses, multiple endpoints are often of interest (either observed or latent), and SEM accommodates these complex structures. As researchers move towards multi-pollutant modeling, SEM is a compelling analytic method that should not be overlooked.

In this paper, we demonstrate how SEM can be used to effectively model the associations between VOCs and respiratory function, controlling for the effects of tobacco smoke exposure on both VOCs and respiratory function in addition to other covariates that influence VOCs and respiratory function separately. Using this analytic approach, we find marginal evidence of association between xylene/ethylbenzene and impaired respiratory function and stronger evidence of association between toluene and impaired respiratory function. We find that poverty is associated with higher levels of toluene exposure, and that poverty is indirectly associated with impaired respiratory function through its relationships with toluene and tobacco smoke exposure.

## Figures and Tables

**Table 1:** Initial distribution of 2011–2012 National Health and Nutritional Examination Surveys (NHANES) variables among youth receiving the volatile organic compound (VOC) supplement (n = 748).

Variable	Label	Num. Missing	Min	Median	Max
PEF	Peak Expiratory Flow Rate (baseline), in mL/s	54	1336	5253	13,437
NEV	Extrapolated Volume (baseline), in mL	54	0	58	201
FVC	Forced Vital Capacity (baseline), in mL	54	592	2658	7205
NUMCIGH	Average number of cigarettes smoked in the home per day	4	0	0	40
NSMOKE	Number of smokers in the household	1	0	0	3
XYLENE1	2-Methylhippuric acid (ng/mL)	23	3.5	24.3	3000.0
XYLENE2	3-methipurc acid & 4-methipurc acid (ng/mL)	23	9.1	173.0	40,100.0
ETHYLBEN	Phenylglyoxylic acid (ng/mL)	23	8.5	189.0	2250.0
TOLUENE	*N*-Acetyl-S-(benzyl)-l-cysteine (ng/mL)	23	0.4	7.2	1180.0
RIDAGEYR	Age in years at screening	0	6	11	18
HEIGHT	Standing Height (dm)	3	10.6	15.0	19.1
WEIGHT	Weight (kg/10)	4	1.6	4.6	17.8
POVERTY	Ratio of family income to poverty	57	0	1.4	5.0

**Table 2:** Weighted Pearson correlation coefficients of observed variables within latent variable measures among youth receiving the VOC supplement (n = 748).

Latent Variable	Observed Variables	Pearson Correlation Coefficient
Tobacco	NUMCIGH, NSMOKE	0.865 ***
Xylene/Ethylbenzene	XYLENE1, XYLENE2	0.692 ***
	XYLENE1, ETHYLBEN	0.172 ***
	XYLENE2, ETHYLBEN	0.235 ***
Respiratory Function	PEF, NEV	0.495 ***
	PEF, FVC	0.877 ***
	NEV, FVC	0.518 ***

*** Correlation significantly different from zero (*α* < 0.0001); Note: Participants missing one or both observed variables were excluded from individual correlation calculations.

**Table 3:** SEM of respiratory function, tobacco smoke exposure, and VOCs: estimates, standard errors, and *p*-values.

Effect	Estimate (*β*)	Standard Error	*p*-Value
RF ^1^ ← Tobacco	–0.012	0.006	0.041
RF ← Xylene/Ethylbenzene	–0.016	0.010	0.120
RF ← Toluene	–0.019	0.010	0.050
RF ← Age	0.065	0.022	0.004
RF ← Height	0.738	0.059	<0.001
RF ← Weight	0.117	0.028	<0.001
Xylene/Ethylbenzene ← Tobacco	0.025	0.018	0.177
Tobacco ← Poverty	–0.646	0.143	<0.001
Xylene/Ethylbenzene ← Poverty	–0.010	0.035	0.781
Toluene ← Poverty	–0.043	0.019	0.023

**^1^** RF = respiratory function.

**Table 4:** Indirect effects of poverty on respiratory function.

Effect	Estimate (*β*)	Standard Error	*p*-Value
Total Indirect	0.009	0.004	0.017
RF ^1^ ← Tobacco ← Poverty	0.008	0.004	0.033
RF ← Toluene ← Poverty	0.001	0.000	0.097
RF ← Xylene/Ethylbenzene ← Poverty	0.000	0.001	0.772
RF ← Xylene/Ethylbenzene ← Tobacco ← Poverty	0.000	0.000	0.320

**^1^** RF = respiratory function.
